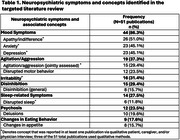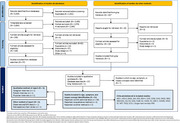# Presence and Impact of Neuropsychiatric Symptoms in Preclinical Alzheimer’s Disease

**DOI:** 10.1002/alz70861_108543

**Published:** 2025-12-23

**Authors:** Zachary Sheff, Kaelyn Rupinski, Ariana Buterbaugh, Madeline Tallarico, E. Jolanda Muenzel, Yuma Yokoi, Nalin Payakachat, Alexandra Atkins

**Affiliations:** ^1^ Eli Lilly & Company, Indianapolis, IN USA; ^2^ Adelphi Values, Boston, MA USA; ^3^ Eli Lilly & Company, Tokyo Japan

## Abstract

**Background:**

Neuropsychiatric symptoms (NPS) in Alzheimer’s disease (AD) are associated with increased caregiver burden, institutionalization, and mortality. Recent evidence indicates that mood, personality, and behavioral changes manifest considerably earlier, often *prior* to observed declines in cognition and functioning.

Understanding the potential value of early interventions on emergence and progression of NPS is needed. This targeted literature review (TLR) aims to characterize NPS in preclinical AD and identify and evaluate clinical outcome assessments (COAs) for NPS in preclinical AD.

**Method:**

This TLR used Cochrane collaboration methodology and the Preferred Reporting Items for Systematic Reviews and Meta‐Analyses (PRISMA) guidelines. The TLR was conducted in Embase, Medline, PsycINFO, EconLit, and EBMR from January 2014 to November 2024. Following abstract and full‐text screening, selected articles were reviewed and NPS concepts were extracted based on relevance to preclinical AD populations (e.g., cognitively unimpaired adults with biomarkers of AD pathology). COAs reported in the selected articles were also extracted and mapped to thematic concepts.

**Result:**

Fifty‐one articles were included (Figure 1). The most frequently reported NPS in preclinical AD included mood symptoms (86.3% of articles reviewed), irritability (31.4%), agitation/aggression (37.3%), sleep‐related symptoms (27.5%), and changes in eating behaviors (17.6%) (Table 1). Of the 20 COAs selected for inclusion in concept mapping, eight measured ≥44% of the key concepts. The most frequently measured sign/symptom theme was mood symptoms, measured by 12 COAs. The Neuropsychiatric Inventory Questionnaire and the Mild Behavioral Impairment Checklist, among others, were common COAs used to assess NPS in preclinical AD.

**Conclusion:**

This TLR found that changes in mood, including apathy, depression, and anxiety are frequently reported in preclinical AD. While less common, agitation and irritability were reported in >30% of studies reviewed. As expected, the reported impact of mood and other NPS on daily function in preclinical AD was low. Additional research can provide insight into selection and implementation of fit‐for‐purpose COAs to evaluate the potential impact of treatment on onset and progression of NPS in preclinical AD.